# Induction-phase infliximab monitoring accelerates time to remission in pediatric IBD: a retrospective cohort study

**DOI:** 10.1093/crocol/otag080

**Published:** 2026-07-20

**Authors:** Nicholas Iovino, Samantha R Paglinco, Mahmoud Abdel-Rasoul, Megan McNicol, Brendan Boyle, Hilary K Michel, Ross M Maltz

**Affiliations:** Division of Pediatric Gastroenterology, Hepatology, and Nutrition, Nationwide Children’s Hospital, Columbus, OH, United States; Department of Pediatric Gastroenterology, St. Christopher’s Hospital for Children, Philadelphia, PA, United States; Center for Biostatistics, Department of Biomedical Informatics, College of Medicine, The Ohio State University, Columbus, OH, United States; Biostatistics Resource, Nationwide Children’s Hospital, Columbus, OH, United States; Department of Pharmacy, Nationwide Children’s Hospital, Columbus, OH, United States; Division of Pediatric Gastroenterology, Hepatology, and Nutrition, Nationwide Children’s Hospital, Columbus, OH, United States; Department of Pediatrics, The Ohio State University Wexner Medical Center, Columbus, OH, United States; Division of Pediatric Gastroenterology, Hepatology, and Nutrition, Nationwide Children’s Hospital, Columbus, OH, United States; Department of Pediatrics, The Ohio State University Wexner Medical Center, Columbus, OH, United States; Division of Pediatric Gastroenterology, Hepatology, and Nutrition, Nationwide Children’s Hospital, Columbus, OH, United States; Department of Pediatrics, The Ohio State University Wexner Medical Center, Columbus, OH, United States

**Keywords:** infliximab, biologic, Crohn’s disease, ulcerative colitis, therapeutic drug monitoring

## Abstract

**Background:**

In pediatric inflammatory bowel disease (IBD), the optimal timing to begin therapeutic drug monitoring (TDM) of infliximab (IFX) is unclear. We hypothesized that TDM during induction of IFX improves clinical outcomes in children with IBD.

**Methods:**

This retrospective cohort study utilized data from an internal database at Nationwide Children’s Hospital between January 1, 2020, and December 31, 2023. Pediatric IBD patients receiving IFX with induction-phase TDM (I-TDM) at Week 6 were compared to those with maintenance-phase TDM (M-TDM) at Week 14. Propensity score matching (PSM) was used for sex, disease, and age at IFX start. The primary outcome was time to clinical remission.

**Results:**

In total, 68 patients were matched from each group. The I-TDM group experienced a shorter median time to clinical remission (140 days [95% CI: 108, 167]) compared to the M-TDM group (203 days [95% CI: 167, 232]; *P* = .006). When adjusting for baseline variations, I-TDM remained independently associated with accelerated clinical remission (aHR = 1.68, 95% CI: 1.10, 2.55; *P* = .016). While time to therapeutic maintenance IFX levels was similar, post hoc analysis showed that 75% of patients in the I-TDM group achieved target levels in 134 days (95% CI: 114, 182) versus 183 days (95% CI: 153, 224) in the M-TDM group. There were no significant differences in long-term complications between the groups.

**Conclusions:**

Induction-phase TDM before the third infusion shortens the time to clinical remission, allowing for more prompt dose adjustments and improving patient outcomes. This approach warrants consideration in clinical practice for children with IBD.

## Graphical Abstract

**Graphical Abstract:**
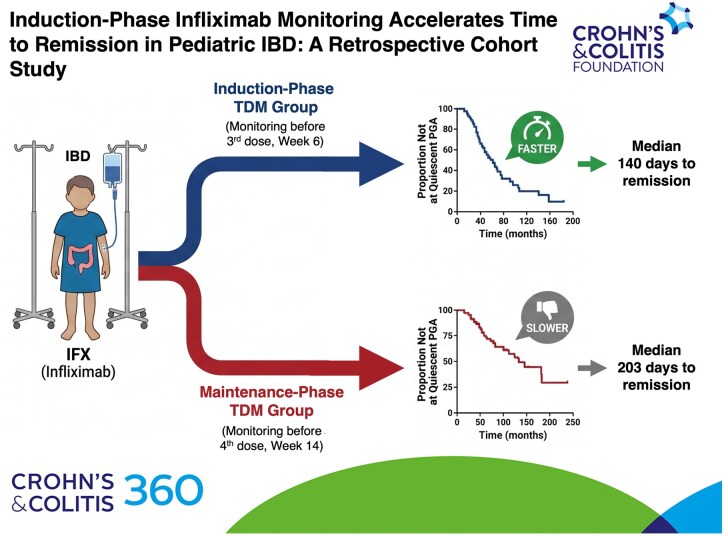

## Introduction

Childhood onset inflammatory bowel disease (IBD) tends to have more extensive disease compared to adults, and children are at risk for linear growth delay, pubertal delay, and bone development deficits.^1–3^ Over time, pediatric Crohn’s disease (CD) often progresses from non-penetrating, non-stricturing inflammatory behavior to stricturing and or penetrating disease.^4^ Similarly, pediatric ulcerative colitis (UC) undergoes extension in about 30% of cases by 5 years and over 50% by 15 years.^5^^,^^6^ Children with IBD therefore endure a unique set of challenges that must be identified and addressed promptly with a multi-modal approach anchored in medical therapy. Early intervention is especially important given the length of diagnostic delay is correlated with bowel stenosis, intestinal surgery, and poor growth and development.^7^^,^^8^ The need for early and effective intervention often necessitates the rapid optimization of advanced medical therapies, such as anti-tumor necrosis factor (anti-TNF) agents.

For pediatric patients with moderate-to-severe IBD, guidelines increasingly support the early initiation of anti-TNF therapy. Infliximab (IFX), a monoclonal antibody against anti-TNF, is a mainstay for children with CD and for children with UC who have not responded to steroids and mesalamines.^9–14^ In a prospective study of children with CD, patients receiving early anti-TNF therapy (started within 90 days of diagnosis) were less likely to develop penetrating disease complications.^15^ Though effective for many, some patients exhibit primary non-response.^16^ Others may experience secondary loss of response (LOR) over time, often due to the development of anti-drug antibodies (immunogenicity) that neutralize the medication.^17^

To mitigate these risks and optimize outcomes, therapeutic drug monitoring (TDM) has become a cornerstone of IFX management. Classic proactive TDM is defined as routine, scheduled measurement of drug concentrations and antibodies in patients who are in stable clinical remission. This contrasts with reactive TDM, which is performed in symptomatic patients. While this can help determine the underlying cause of treatment failure (eg, low drug level or development of anti-drug antibodies) and guide subsequent treatment decisions (eg, increasing IFX dose or frequency or switching to another biologic), this delayed approach often fails to prevent LOR or immunogenicity.^18–20^

In pediatric cohorts, initiating scheduled TDM during the transition from the induction phase to the maintenance phase is a critical decision point. Following the standard IFX induction at Weeks 0, 2, and 6, the trough level prior to the fourth dose at Week 14 marks the formal transition to the maintenance phase. Both adult and pediatric studies have consistently shown that higher IFX trough levels are strongly associated with improved clinical outcomes, providing a clear rationale for early dose optimization.^21–28^

Evidence from landmark clinical trials reinforces the benefits of scheduled TDM. For example, a randomized controlled trial from Vande Casteele et al. (TAXIT study) found that adult patients who underwent scheduled TDM had significantly fewer flares compared to patients undergoing clinic-based TDM.^27^ Long-term follow up of the TAXIT cohort found that patients from the scheduled TDM group also experienced a lower rate of IFX discontinuation within 1 year.^29^ Current consensus guidelines, including the recently published NASPGHAN position paper on TDM, recommend scheduled TDM with target trough levels ≥18 μg/mL at Week 6 (prior to the third infusion) and ≥7 μg/mL at Week 14 (prior to the fourth infusion).^21–23^^,^^25^^,^^30^^,^^31^ Importantly, while Week 14 represents the first steady-state trough and has been a widely adopted standard of care for initiating maintenance-phase TDM, there is growing interest in initiating TDM even earlier, during the induction phase.

There are several pediatric studies suggesting that IFX trough levels measured prior to the third infusion at Week 6 are associated with both early clinical response and sustained remission. In their retrospective observational cohort study, Ungar et al. found a positive association between higher Week 6 trough levels and clinical remission beyond 1 year.^32^ A later study from Clarkston et al. found that IFX concentrations at Week 6 (≥18 µg/mL) were associated with improved early clinical outcomes at Week 14 and achieving target levels for the maintenance phase (≥5 µg/mL).^33^ Most recently, a Dutch pediatric retrospective study found that patients who sustained remission between 26- and 52-weeks post-induction had higher IFX levels at Week 6 and Week 14.^34^ However, to our knowledge, no prior studies have directly compared clinical outcomes in children with IBD who utilized scheduled TDM at Week 6 versus Week 14.

Following an internal quality improvement (QI) initiative at Nationwide Children’s Hospital (NCH), our IBD center transitioned from starting scheduled TDM at Week 14 to Week 6 as the standard of care. This change in practice forms the basis of our study, which compares induction-phase TDM (initiated at Week 6 prior to the third infusion) with maintenance-phase TDM (initiated at Week 14 prior to the fourth infusion). We hypothesize that induction-phase TDM (I-TDM) accelerates time to remission, shortens the time to achieve therapeutic drug levels, decreases LOR, and reduces IFX discontinuation compared to maintenance-phase TDM (M-TDM).

## Materials and methods

### Population and study design

This single-center retrospective cohort study included patients diagnosed with CD, UC, and IBD-unclassified (IBD-U) aged 2 to 21 years and started on IFX between January 1, 2020, and December 31, 2023. Patients with IBD-U were grouped with UC for analysis. Patients were excluded if they received fewer than four IFX infusions. The I-TDM group was defined as patients who had IFX trough levels first drawn prior to the third infusion (at 6 weeks ± 7 days). The M-TDM group was defined as patients who had IFX trough levels first drawn prior to the fourth infusion, depending on clinical judgment of providers. All patients were identified from the Center for Pediatric and Adolescent IBD internal database at NCH, a tertiary center in Columbus, Ohio. Adjustment of infliximab dosing or frequency and timing of follow-up visits were at physician discretion. The IBD center recommended dosing adjustments if the IFX level prior to the third infusion was ≤18 µg/mL and repeating another level after two infusions at the new dosage. If the level was ≥18 µg/mL, it was advised to obtain another level prior to the fourth infusion. If IFX levels were ≤5 µg/mL during maintenance therapy, the IBD center recommended adjusting the dose or frequency.

### Data quality and procedures

Study participants were identified from new IFX starts within the NCH IBD database. Data were manually cleaned to ensure accuracy of infusion dates and laboratory timestamps. The investigators had full access to the clinical database for the duration of the study period. No person- or institution-level linkages were performed.

### Patient demographics

Patient demographics, including weight, height, body mass index (BMI) z-scores, disease characteristics and behavior (Paris classification),^35^ IFX dosing and intervals, and laboratory values (albumin [Alb], hemoglobin [Hgb], erythrocyte sedimentation rate [ESR], C‐reactive protein [CRP], fecal calprotectin) were collected at baseline (time of IFX initiation), 6 months (±1.5 months), and 12 months (± 2 months) post-IFX initiation. In addition, IFX trough levels, anti‐drug antibody levels, physician global assessment (PGA), and dose adjustments were documented throughout the first 12 months following IFX initiation. Serum IFX concentrations and anti-drug antibodies were measured using a cell-based functional bioassay (ARUP Laboratories, Salt Lake City, UT), which functions as a drug-sensitive assay.

### Outcomes

The primary outcome of this study was time to clinical remission, which was defined as the first recorded quiescent PGA score from an office visit. Secondary outcomes included time to therapeutic maintenance IFX levels, and rates of IBD-related hospitalizations, IBD-related surgeries, and IFX discontinuation, and clinical outcomes (PGA, IBD-related hospitalization or surgery, IFX discontinuation, serum laboratory markers, or anthropometrics) at 6- and 12-month post-IFX initiation for patients in both groups. IBD-related surgeries included colectomy, small bowel resection, or fistulotomy. Therapeutic maintenance IFX levels were defined as ≥5 μg/mL. A maintenance IFX level was defined as an IFX level measured at or after the fourth infusion.

### Statistical analysis

This study employed a propensity score matching (PSM) methodology to identify a 1:1 matched cohort of included patients based on sex, disease (CD, UC/IBD-U), and age at IFX initiation (±2 years). Logistic regression modeling was used to estimate propensity scores, and greedy nearest-neighbor matching with a caliper distance of 0.5 was used to obtain the 1:1 matched cohort. Patients without a match were excluded. Baseline demographics of matched cohorts are summarized as frequencies (%) for categorical variables and as medians with interquartile range (IQR) for continuous variables. Chi-squared or Fisher’s exact tests was utilized for categorical variables. Linear mixed effect models were fit to estimate and compare continuous variables, which included laboratory values (Alb, Hgb, ESR, CRP, and fecal calprotectin) and z-scores (height, weight, and BMI). The two-tailed Mann-Whitney U Test was used for comparison of starting IFX dosing between the I-TDM and M-TDM groups.

Kaplan-Meier plots and log-rank tests were used for comparison of time-to-event outcomes, including time to clinical remission, time to therapeutic maintenance IFX levels, and time to IBD-related hospitalization, surgery or therapy discontinuation. To account for baseline clinical, demographic, and biochemical differences, multivariable Cox proportional hazards regression modeling was performed within the matched cohort. The Cox models adjusted for age, sex, initial dose, disease phenotype, and baseline laboratory markers, including albumin, hemoglobin, ESR, and CRP. Fecal calprotectin was not included due to limited availability of baseline data. Observations missing baseline laboratory metrics were omitted, allowing for complete sub-analysis of observations.

For longitudinal continuous outcomes, linear mixed effects models included random intercepts to account for correlation of repeated measures. These models included main effects for treatment group (I-TDM vs M-TDM), disease type (CD and UC/IBD-U), time (baseline, 6 and 12 months), and the interaction between treatment group and time. Hypothesis testing was two-sided and conducted at an alpha of 0.05. All statistical analyses were conducted using SAS version 9.4 (SAS Institute, Cary NC).

## Ethical considerations

Institutional Review Board approval was obtained from NCH in Columbus, Ohio (STUDY00003324).

## Results

### Study population

In total, 90 patients were identified in the I-TDM group and 83 patients were identified in the M-TDM group. Using PSM methodology, 68 patients from each group were matched (Table 1). The median age at diagnosis, median age at IFX initiation, sex, race, and CD or UC/IBD-U phenotype and behavior were similar between the groups (Table 1). At IFX initiation, laboratory values (albumin, Hgb, CRP, ESR, and calprotectin) and growth factors (weight, height, and BMI) were similar between the groups. More patients from the I-TDM group (47%) had a higher starting IFX dose (10 mg/kg) compared to patients of the M-TDM group (31%), however, the difference was not statistically significant (*P* > .05) (Table 1).

**Table 1:** Demographics of patients by group at infliximab initiation.

	Induction-phase TDM (*n* = 68)	Maintenance-phase TDM (*n* = 68)	*P* value
**Disease, *n* (%)**			
** CD**	50 (74%)	50 (74%)	1.00^b^
** UC/IBD-U**	18 (26%)	18 (26%)	
**Age of diagnosis (years), med (IQR)**	14.8 (12.0 to 17.0)	15.5 (12.0 to 17.8)	.28^a^
**Age of first IFX dose (years), med (IQR)**	15.1 (12.8 to 17.4)	16.2 (13.0 to 18.2)	.06^a^
**Sex, *n* (%)**			
** Female**	25 (36.8%)	25 (36.8%)	1.00^b^
**Race, *n* (%)**			
** Asian**	2 (2.9%)	1 (1.5%)	.15^b^
** Black**	10 (14.7%)	12 (17.6%)	
** Multi-racial**	0 (0.0%)	5 (7.4%)	
** Unknown**	1 (1.5%)	0 (0.0%)	
** White**	55 (80.9%)	50 (73.5%)	
**Weight (z-score), med (IQR)**	−0.4 (−1.1 to 0.4)	−0.6 (−1.3 to 0.7)	.99^a^
**Height (z-score), med (IQR)**	0.0 (−0.7 to 0.7)	−0.1 (−0.8 to 0.6)	.44^a^
**BMI (z-score), med (IQR)**	−0.6 (−1.4 to 0.2)	−0.5 (−1.6 to 0.6)	.83^a^
**CD Location, *n* (%)**			.11^b^
***Lower GI (n = 99)***			
** L1**	11 (69%)	5 (31%)	
** L2**	9 (36%)	5 (64%)	
** L3**	30 (43%)	39 (57%)	
***Upper GI (n = 84)***			
** L4a**	25 (48%)	27 (52%)	
** L4b**	18 (56%)	14 (44%)	
**CD behavior, *n* (%)**			.14^b^
** B1**	38 (76%)	41 (82%)	
** B2**	1 (2%)	4 (8%)	
** B3**	3 (6%)	6 (6%)	
** B2/B3**	2 (4%)	10 (10%)	
**Perianal involvement, *n* (%)**	9 (18%)	14 (28%)	.23^b^
**UC Disease extension, *n* (%)**			.29^b^
** E1**	1 (5.6%)	0 (0%)	
** E2**	2 (11.1%)	4 (22.2%)	
** E3**	2 (11.1%)	0 (0%)	
** E4**	13 (72.2%)	14 (77.8%)	
**Labs, med (IQR)**			
** Alb (g/dL)**	4.0 (3.3 to 4.3)	3.7 (3.2 to 4.2)	.25^a^
** Hgb (g/dL)**	11.6 (10.2 to 13.1)	11.4 (10.2 to 12.6)	.43^a^
** CRP (mg/dL)**	1.3 (0.5 to 2.9)	1.8 (0.5 to 3.6)	.44^a^
** ESR (mm/hr)**	29 (10.0 to 45.0)	27 (10.0 to 48.0)	.78^a^
** Calprotectin (µg/g)**	2221 (1117.0 to 3000.1)	2179 (1202.0 to 3000.1)	.71^a^

Abbreviations: Alb, albumin; BMI, body-mass index; CD, Crohn’s disease; CRP, C-reactive protein; ESR, sedimentation rate; Hgb, hemoglobin; IBD-U, inflammatory bowel disease unclassified; IFX, infliximab; IQR, interquartile range; med, median; TDM, therapeutic drug monitoring; UC, ulcerative colitis.

a Kruskal-Wallis *P*-value.

b Chi-square *P*-value.

### Time to clinical remission and therapeutic infliximab levels

**Figure 1:**
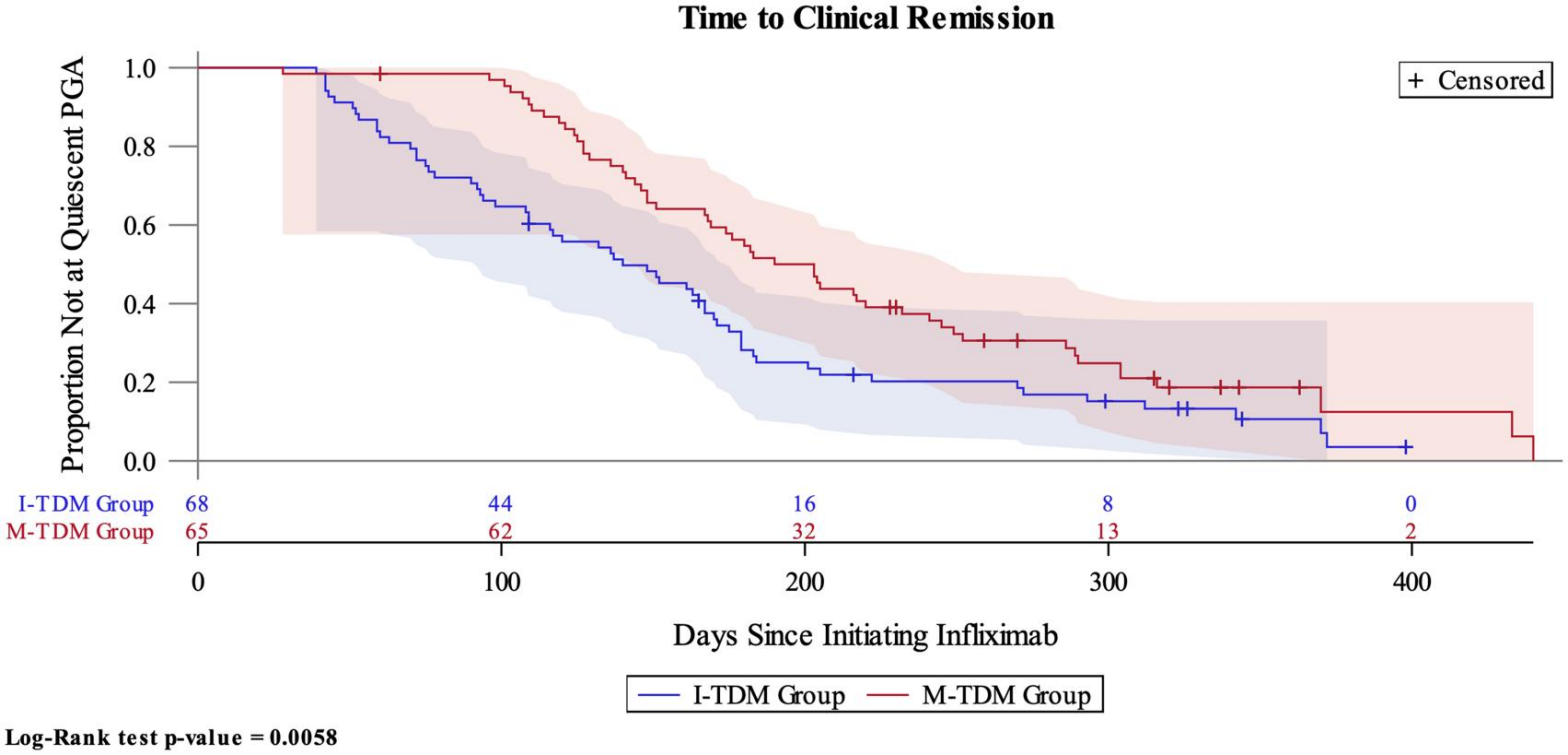
Time to clinical remission (quiescent Physician Global Assessment score) using Kaplan-Meier was fastrer in the I-TDM group versus M-TDM group. I-TDM, induction-phase therapeutic drug monitoring; M-TDM, maintenance-phase therapeutic drug monitoring.

Patients from the I-TDM group achieved clinical remission in a median of 140 days (95% CI: 108, 167) versus 203 days (95% CI: 167, 232) in patients from the M-TDM group (Figure 1, *P* = .006). To isolate the effect of TDM timing from differences in baseline dosages, multivariable Cox proportional hazards regression analysis adjusting for baseline starting dose was performed. In the univariable analysis (Table 2, Model 1), I-TDM was associated with a significantly faster time to clinical remission (HR = 1.68, 95% CI: 1.15, 2.45, *P* = .007). This relationship remained highly significant when adjusting for starting dose categorically (aHR = 1.717, 95% CI: 1.17, 2.52, *P* = .006) or continuously (aHR = 1.67, 95% CI: 1.14, 2.43, *P* = .008).

**Table 2:** Cox proportional hazards regression models for time to clinical remission.

	Hazard ratio	95% confidence interval	*P* value
**Model 1 (*n* = 133)**			
** I-TDM**	1.68	1.16, 2.45	**.007** ^a^
**Model 2 (*n* = 133)**			
** I-TDM**	1.59	1.09, 2.33	**.017** ^a^
** Age**	0.95	0.90, 1.01	.11^a^
** Sex**	0.98	0.67, 1.45	.93^a^
** IFX dose**	1.02	0.97, 1.07	.53^b^
**Model 3 (*n* = 113)**			
** I-TDM**	1.68	1.10, 2.55	**.016** ^a^
** Age**	0.95	0.89, 1.02	.18^b^
** Sex**	1.15	0.73, 1.81	.55^a^
** Disease phenotype**	1.02	0.95, 1.10	.53^a^
** IFX dose**	1.36	0.80, 2.31	.26^b^
** Alb (g/dL)**	0.99	0.64, 1.54	.97^b^
** Hgb (g/dL)**	1.02	0.86, 1.20	.81^b^
** CRP (mg/dL)**	0.97	0.88, 1.06	.45^b^
** ESR (mm/h)**	1.00	0.99, 1.01	.59^b^

Note: Model 1: univariable analysis; Model 2: expanded model; Model 3: comprehensive model.

Abbreviations: Alb, albumin; CRP, C-reactive protein; ESR, sedimentation rate; Hgb, hemoglobin; IFX, infliximab; I-TDM, induction-phase therapeutic drug monitoring.

a Type 3 Wald *P*-value.

b Covariate Wald *P*-value.

Further, adjusting for demographics and initial dose simultaneously in an expanded model (Table 2, Model 2) preserved the significant independent effect of early monitoring (aHR = 1.59, 95% CI: 1.09, 2.33, *P* = .017). Finally, a comprehensive model was completed (*n* = 116) which added disease phenotype and baseline laboratory values (Table 2, Model 3). After controlling for age, sex, disease phenotype, initial dose, and laboratory values (albumin, Hgb, CRP, ESR), I-TDM remained independently associated with accelerated time to clinical remission (aHR = 1.68, 95% CI: 1.10, 2.55, *P* = .016).

**Figure 2:**
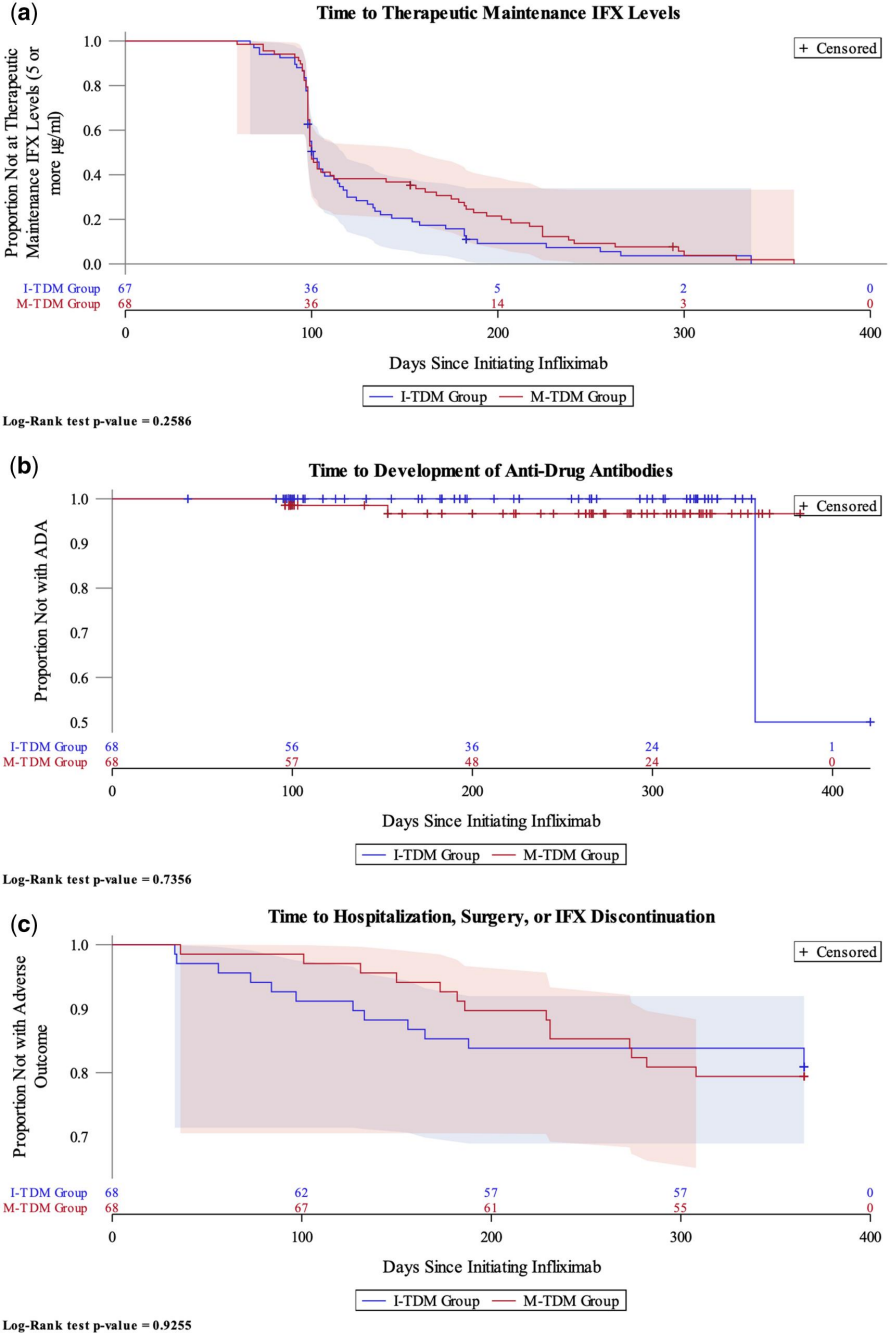
Association of time to (a) therapeutic maintenance IFX level no difference between I-TDM vs M-TDM, (b) development of anti-drug antibodies no difference between I-TDM vs M-TDM, and (c) hospitalization, surgery, or therapy discontinuation no difference between I-TDM vs M-TDM using Kaplan-Meier. Kaplan Meier graphs for survival are according to study group (I-TDM group vs M-TDM group). IFX, infliximab; I-TDM, induction-phase therapeutic drug monitoring; M-TDM, maintenance-phase therapeutic drug monitoring.

There were no differences in time to therapeutic maintenance IFX levels or development of anti-drug antibodies between the groups (Figure 2A and B, *P* > .05). However, post hoc analysis showed 75% of patients from the I-TDM group had achieved therapeutic maintenance IFX levels (≥5 μg/mL) by 134 days (95% CI: 114, 182) versus 183 days (95% CI: 153, 224) for 75% of the M-TDM group to achieve target levels.

### Infliximab trough levels

At Week 6, patients from the I-TDM group had a median IFX level of 25.2 μg/mL (IQR 14.6-40.1), with 70% (*n* = 48) having an IFX level of ≥18 µg/mL. Of the patients from the I-TDM group who had levels re-measured prior to the fourth infusion (*n* = 48), median IFX levels was 11.8 (IQR 5.0-16.9) compared to 7.2 μg/mL (IQR 1.6-12.7) in patients from the M-TDM group (*P* < .013). Prior to the fourth infusion, 75% (*n* = 35) of patients from the I-TDM group had levels ≥5 µg/mL versus 61% (*n* = 41) in the M-TDM group. The median time at which levels were first measured in the M-TDM group was 98 days (IQR 96-100) versus 98 days (IQR 97-106) for patients in the I-TDM group who had levels re-measured prior to the fourth infusion.

### Clinical outcomes at 6 and 12 months

There were no significant differences in the IFX dosing between the two groups at 6 and 12 months (Table 3). There were no differences in time to or number of IBD-related hospitalizations (6 I-TDM vs 4 M-TDM), IBD-related surgeries (4 I-TDM vs 1 M-TDM), IFX discontinuation (6 I-TDM vs 7 M-TDM), between the groups (Figure 2C  *P* > .05). There were no significant differences in laboratory markers (Alb, Hgb, CRP, ESR, calprotectin, and IFX levels) at 6 or 12 months between the groups (*P* > .05; Table 3). In addition, there were no differences in weight, height, BMI z-score, or PGA between the groups at 6 or 12 months (*P* > .05; Table 3).

**Table 3:** Outcomes and levels at 6 and 12 months.

	Induction-phase TDM (*n* = 68)	Maintenance-phase TDM (*n* = 68)	*P* value
**Hospitalizations, *n* (%)**	8 (11.8%)	9 (13.2%)	.79^b^
** Length (days)**	2.0 (1.0 to 6.0)	10.0 (3.0 to 38.0)	.18^a^
**Surgery, *n* (%)**	4 (5.9%)	2 (2.9%)	.40^b^
**Therapy stopped, *n* (%)**	6 (8.8%)	8 (11.8%)	.57^b^
**Antibodies, *n* (%)**	2 (3.7%)	0 (0.0%)	.13^b^
**6-month, med (IQR)**			
** Weight (z-score)**	0.1 (−0.6 to 0.7)	−0.2 (−0.8 to 0.9)	.65^a^
** Height (z-score)**	−0.2 (−0.8 to 0.8)	−0.1 (−0.7 to 0.6)	.88^a^
** BMI (z-score)**	0.1 (−0.6 to 0.7)	0.1 (−0.8 to 0.8)	.63^a^
** IFX (µg/dL)**	12.7 (4.9 to 18.9)	13.2 (5.6 to 19.0)	.89^a^
** Alb (g/dL)**	4.4 (4.2 to 4.6)	4.3 (4.1 to 4.5)	.28^a^
** Hgb (g/dL)**	13.4 (12.3 to 14.4)	13.4 (12.3 to 14.4)	.79^a^
** CRP (mg/dL)**	0.5 (0.5 to 0.6)	0.5 (0.5 to 0.5)	.63^a^
** ESR (mm/hr)**	10.0 (5.0 to 20.0)	10.0 (4.0 to 18.5)	.56^a^
** Calprotectin (µg/g)**	230.5 (79.5 to 532.5)	230.5 (79.5 to 532.5)	.57^a^
** 6-month PGA, *n* (%)**			.79^b^
** Quiescent**	34 (77.3%)	32 (71.1%)	
** Mild**	8 (18.2%)	10 (22.2%)	
** Moderate**	2 (4.5%)	3 (6.7%)	
** Severe**	0 (0.0%)	0 (0.0%)	
**12-month, med (IQR)**			
** Weight (z-score)**	0.2 (−0.6 to 0.6)	0.2 (−0.7 to 0.9)	.86^a^
** Height (z-score)**	0.0 (−0.7 to 0.7)	−0.1 (−0.6 to 0.6)	.61^a^
** BMI (z-score)**	0.0 (−0.5 to 0.7)	0.0 (−0.8 to 0.7)	.81^a^
** IFX (µg/dL)**	13.9 (6.9 to 24.5)	12.8 (8.6-16.5)	.57^a^
** Alb (g/dL)**	4.4 (4.3 to 4.6)	4.4 (4.2 to 4.6)	.73^a^
** Hgb (g/dL)**	13.5 (12.6 to 14.4)	13.4 (12.6 to 14.4)	.80^a^
** CRP (mg/dL)**	0.5 (0.5 to 0.6)	0.5 (0.5 to 0.5)	.42^a^
** ESR (mm/h)**	10.0 (6.0 to 19.0)	10.0 (4.0 to 17.0)	.34^a^
** Calprotectin (µg/g)**	315.0 (35.0 to 811.0)	413.0 (56.0 to 1315.0)	.53^a^
**12-month PGA, *n* (%)**			.75^b^
** Quiescent**	42 (85.7%)	36 (81.8%)	
** Mild**	4 (8.2%)	4 (9.1%)	
** Moderate**	3 (6.1%)	3 (6.8%)	
** Severe**	0 (0.0%)	1 (2.3%)	

Abbreviations: Alb, albumin; BMI, body-mass index; CRP, C-reactive protein; ESR, sedimentation rate; Hgb, hemoglobin; IFX, infliximab; IQR, interquartile range; med, median; PGA, Physician Global Assessment; TDM, therapeutic drug monitoring.

a Kruskal-Wallis *P*-value.

b Chi-square *P*-value.

## Discussion

In our pediatric cohort, we found that I-TDM at Week 6 was associated with faster time to clinical remission for children with IBD compared to M-TDM at Week 14. Although we found no difference in long-term clinical or biochemical outcomes at 6 and 12 months, achieving faster remission is clinically meaningful for pediatric patients. Childhood-onset disease is uniquely aggressive, subjecting patients to growth delays, extensive school absenteeism, and psychosocial anxiety.^36–38^ Furthermore, pediatric IBD imposes major financial and emotional burdens on caregivers through work impairment and out-of-pocket costs.^39–41^ Rapidly optimizing therapy to shorten the duration of active symptomatic disease minimizes these far-reaching complications and provides an earlier window to restore quality of life for both patients and caregivers.

Our study builds upon a growing body of evidence emphasizing the importance of high induction anti-TNF concentrations. Post hoc analysis of the ACT-1 and ACT-2 trials in adults with UC found that higher IFX levels at Week 8 correlate with clinical remission and mucosal healing.^22^ Similarly, in pediatric cohorts, higher IFX target levels (ranging from 8.3 to 18 μg/mL) during induction have been associated with improved early and long-term outcomes.^25^^,^^33^^,^^34^ Most recently, the NASPGHAN position paper on TDM recommends targeting trough IFX levels ≥18 μg/mL at Week 6 and ≥7 μg/mL at Week 14.^30^ However, while these guidelines establish a clear precedent for target thresholds, the position paper does not provide a recommendation on the optimal timing for initiating TDM. This ambiguity highlights a major gap in practice addressed by our current study.

While induction trough levels were not measured in the M-TDM group (preventing a direct baseline comparison), patients from the I-TDM group had significantly higher IFX levels by the time of the fourth infusion at Week 14 (11.8 vs 7.2 µg/mL, *P* < .05). This difference in drug exposure at Week 14 likely represents an expected result of dose adjustments made at Week 6. Importantly, while more patients in the I-TDM group received a higher initial starting dose of 10 mg/kg compared to the M-TDM group (47% vs 31%), adjusting for baseline starting dose via multivariable Cox regression modeling confirmed the independent effect of I-TDM. Further, the association between I-TDM and accelerated time to clinical remission remained significant even when broadly adjusting for initial dose, demographics, disease phenotype, and baseline laboratory markers. These findings provide evidence that the accelerated remission observed in the I-TDM group is likely driven by earlier IFX dose optimization rather than baseline differences in dosing.

These findings can be explained by the unique pharmacologic properties of pediatric patients. Compared to adults, children clear IFX more rapidly due to higher systemic inflammatory burden and lower serum albumin.^42–44^ This translates to higher vulnerability during induction and priming of the immune system to generate anti-drug antibodies (ADAs), leading to immune-mediated pharmacokinetic failure. While our cohort had a low rate of immunogenicity, I-TDM allows for prompt dose adjustments to counteract rapid clearance, helping patients achieve therapeutic targets faster and avoiding prolonged sub-therapeutic exposure that could otherwise lead to loss of response.

Induction-phase TDM may also prove particularly useful for optimizing a subset of patients who exhibit more rapid clearance. While the median time to achieve therapeutic maintenance IFX levels was similar, 75% of patients in the I-TDM group reached target levels by Day 134 compared to Day 183 in the M-TDM group. This post hoc observation suggests that early monitoring effectively rescues rapid clearers who would have otherwise spent an additional 7 weeks in a sub-therapeutic range prior to their first scheduled level check at Week 14. Once the M-TDM group underwent their first TDM-guided optimization at Week 14, their clinical trajectories converged with the I-TDM group, explaining the lack of statistical differences in hospitalizations, surgeries, or drug discontinuation rates 6 and 12 months.

The present study has several limitations. First, because parameters such as baseline corticosteroid, immunomodulator, and nutritional of utilization therapies were not routinely recorded within our registry during the study period, we were unable to account for these potential therapeutic confounders. Second, because our center utilized a drug-sensitive functional neutralizing antibody assay, antibody detection may have been masked in patients with high systemic drug concentrations. Moreover, our primary outcome relied on PGA alone for defining clinical remission (rather than PUCAI, sPCDAI, or endoscopic data) which may be subject to inter-provider variability. Patient management and follow-up were also non-standardized and at the discretion of the provider and could have therefore introduced detection bias. Lastly, a cost-benefit analysis was not performed. However, current model care guidelines note that induction-phase TDM is likely to be cost-effective in children by preventing disease complications, reducing hospitalizations, and avoiding costly biologic class-switching.^30^

## Conclusion

This study demonstrates a clear benefit to initiating induction-phase TDM prior to the third infusion at Week 6. Measuring IFX levels earlier was independently associated with faster time to clinical remission and therapeutic maintenance IFX levels compared with maintenance-phase TDM. Although long-term outcomes converge at 1 year, a 2-month reduction in active, symptomatic disease during the formative years of childhood represents a meaningful improvement in quality of life for both pediatric patients and their caregivers. While future prospective studies utilizing objective endoscopic endpoints are needed to confirm these findings, our study suggests that a simple shift to induction-phase TDM is a highly effective optimization strategy that should be strongly considered across pediatric IBD practice.

## Data Availability

Data for this study is not publicly available.
